# Exceptionally robust magnetism and structure of SrFeO$_2$ above 100 GPa

**DOI:** 10.1038/s41598-022-20192-w

**Published:** 2022-09-26

**Authors:** V. Balédent, L. Nataf, J.-P. Rueff

**Affiliations:** 1 Université Paris-Saclay, CNRS, Laboratoire de Physique des Solides, 91405 Orsay, France; 2 Synchrotron SOLEIL, L’Orme des Merisiers, BP 48 St Aubin, 91192 Gif-sur-Yvette, France; 3 Sorbonne Université, CNRS, Laboratoire de Chimie Physique–Matière et Rayonnement, LCPMR, 75005 Paris, France

**Keywords:** Electronic properties and materials, Structure of solids and liquids

## Abstract

We report the exceptional structural and magnetic stability of SrFeO$_2$ under pressure by X-Ray Magnetic Circular Dichroism (XMCD) and X-ray Diffraction (XRD) up to the Mbar range. The XMCD data confirm the onset of ferromagnetism above 30 GPa and its stability up to 102 GPa while XRD shows that SrFeO$_2$ structure remains unchanged from 30 GPa up to 111 GPa without any sign of structural transition. Our results demonstrate the robustness of Fe properties under extreme conditions in the square planar environment.

## Introduction

The investigation of the stability of iron in different oxygen environments under high pressure has attracted strong interest because of its broad impact for research in chemistry to geophysics. Understanding how iron behaves at extreme conditions, in particular its valence and spin state, can help to explain the stability of various Fe phases, guide the synthesis of new resistant materials and test theoretical approaches of Fe electronic structure at extreme conditions^1^. The crystalline structures of iron oxides and their stability under high pressure is especially important to understand the formation and dynamics of Earth-like planet interiors which are rich in Fe-bearing minerals^2,3^. For example, Fe$_2$O$_3$ undergoes a series of transitions in the 0-100 GPa range with 5 different structures in the 40–50 GPa region^4^. Above the last transition pressure at 50 GPa, no long range order is detected^5^. In Fe$_3$O$_4$, another very common form of iron oxide, a structural transition occurs at 8 GPa^6^ while the ferromagnetic state progressively disappears under pressure, vanishing at 70 GPa^7^. Other less common iron oxides like Fe$_4$O$_5$^8^ and Fe$_5$O$_6$ were recently discovered to be stable under pressure, but decompose into FeO and Fe$_3$O$_4$ above 40 GPa^9^. Finally, FeO is expected to have a zero spin configuration above 70 GPa^10^.

In all these compounds, Fe atoms occupy exclusively octahedral or tetrahedral sites. Recently a new system with iron in a square planar oxygen environment, SrFeO$_2$, was synthesized^11^, offering a new playground to investigate the magnetic and structural stability of Fe in a different local symmetry. SrFeO$_2$ crystallized in the *P*4/*mmm* space group with Fe-O forming planar layers sandwiched by Sr atoms. The magnetic and structural properties of SrFeO$_2$ under pressure were previously studied by Mössbauer spectroscopy, resistivity and X-ray diffraction^12^, and subsequently by X-ray emission spectroscopy^13^ up to 40–50 GPa. In this pressure range, SrFeO$_2$ is shown to undergo a series of well-identified electronic, magnetic and structural transitions. The main change occurs around 40 GPa with a sudden contraction of the lattice within the same space group, a decrease of the magnetic moment and drop of the resistivity, which marks a transition from an antiferromagnetic, insulating high spin state (AFM-I-HS) to ferromagnetic, metallic intermediate spin state (FM-M-IS). Additional phenomena are expected at higher pressure from the details of the molecular orbitals^14^ but data are scarce in this pressure range. Resistivity measurements reveal an anomaly between 65 and 90 GPa which is interpreted as a resurgence of metal-insulator transition^15^. On the other hand, DFT calculations^16^ predict that magnetism will survive well within the Mbar range within the same structure but data are lacking. In this article, we explore the structural and magnetic stability of SrFeO$_2$ above 100 GPa by X-ray diffraction (XRD), X-ray absorption spectroscopy (XAS) and X-ray circular magnetic dichroism (XMCD) at the Fe K-edge. The results demonstrate the outstanding stability of SrFeO$_2$ up to 110 GPa.

## Results

**Figure 1:**
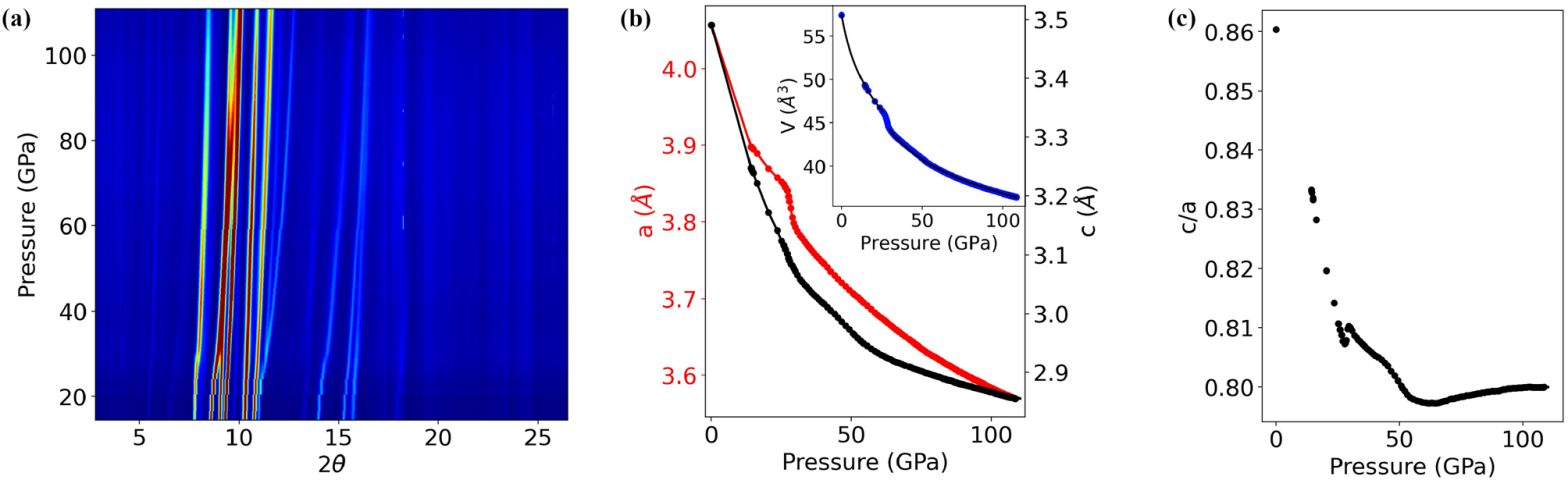
(**a**) Color map of the diffractogram as function of pressure. (**b**) Evolution of the lattice parameters *a* (red circles, left scale) and *c* (black circles, right scale) as a function of pressure; the typical error bars for both parameters is around 0.002 Å, smaller than the point size; inset: evolution of the volume as a function of pressure (blue circles) and fit (black lines) using a 3rd order Birch-Murnaghan EOS. Error bar for the volume is less than 0.1Å$^3$, smaller than the point size. (**c**) Evolution of the *c*/*a* ratio as a function of pressure.

The diffractograms collected between 13 and 111 GPa are presented as a color map in Fig. 1a. The intensity is displayed in logarithmic scale to enhance the weak intensity features. The change in structure is clearly visible around 30 GPa. Using a Le Bail fit on the 19 first Bragg reflections, we extracted the values of the *a* and *c* lattice parameters. Their evolution as function of pressure is shown in Fig. 1b along with the *c*/*a* ratio in Fig. 1c. The structural transition found at $P_c \approx 30$ GPa is characterized by a sizable contraction of the *a* parameter while the *c* parameter exhibits only a slope change marking a variation of its compressibility. The overall result is consistent with previous diffraction experiment reported^12^ up to 45 GPa. The evolution of the volume represented in the inset of Fig. 1b has been fitted independently in the two different pressure ranges on both sides of $P_c$ using a third order Birch-Murnaghan equation of state (EOS). The obtained bulk modulus $B_0$ and its derivative with respect to pressure $B_0'$ are $B_0$= 53±1 GPa, $B_0'$=8±1 GPa for the low pressure regime, and $B_0$= 168±2 GPa, $B_0'$=10±1 GPa for the high pressure regime. No sign of any structural change is visible at high pressure up to 111 GPa, demonstrating the remarkable structural stability of SrFeO$_2$ over a wide range of pressure. A close look at the *c*/*a* ratio in Fig. 1c nevertheless indicates a slope change in the compressibility starting around 60 GPa which could be reminiscent of the anomaly found in resistivity data in a similar pressure range^15^.

**Figure 2:**
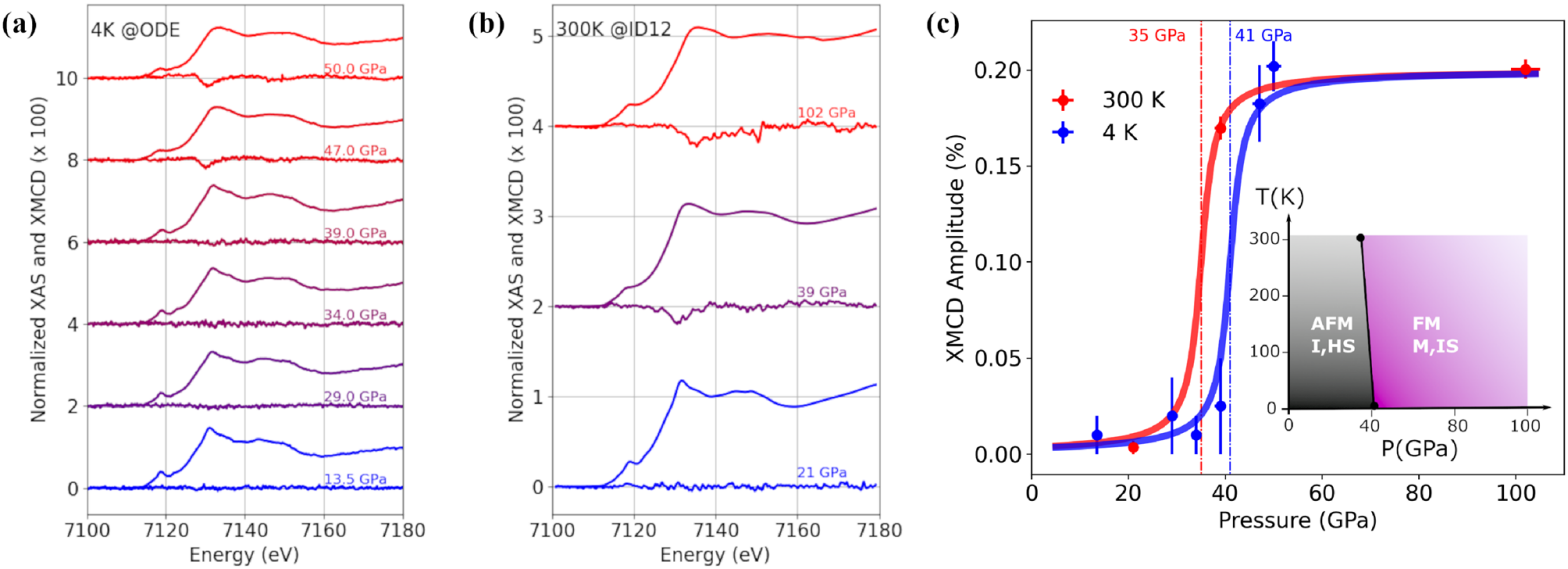
XAS and XMCD spectra for increasing pressure from bottom to top measured at 4 K (**a**) and 300 K (**b**); (**c**): XMCD amplitude at 4 K (blue circles) and 300 K (red circles); thick lines are guides to the eyes and dashed lines mark the AFM—FM pressure transition $P_m$; the inset shows a schematic phase diagram of the AFM and FM phases.

XAS and XMCD spectra at 4K and 300 K are reported in Fig. 2a,b for several pressures from 13.5 to 102 GPa and the XMCD amplitude in Fig. 2c. As observed in Fig. 2, no XMCD signal was measured at 21 GPa within the error bar as expected from the AFM structure. Above the structural transition ($P > 30$ GPa), the XMCD signal progressively builds up reaching full intensity around 50 GPa as shown in Fig. 2c. This signal confirms the FM state of the IS, $S=1$ high pressure phase which was reported previously^12,13^. At 4K, the dichroic signal is absent up to 39 GPa and becomes finite at 47 GPa with the same amplitude as at the ambient temperature XMCD. The absence of dichroic signal at 39 GPa at 4 K suggests that the magnetic transition pressure $P_m$ shifts slightly towards high pressure with decreasing temperature as illustrated by the guides in Fig. 2c. From Fig. 2c, we can estimate $P_m$ at 35 GPa and 41 GPa at 300 K and 4 K respectively. Such an increase of $P_m$ at low temperature is a feature commonly found in Fe magnetic materials including elemental Fe^17^. At 102 GPa, 4K both XAS and XMCD spectra are similar to the 39 GPa, 4K pressure point with a comparable dichroic amplitude. In addition to the structural stability, the spectroscopic results further reveal the exceptional robustness of both electronic and magnetic states.

## Discussion and conclusion

The results demonstrate that SrFeO$_2$ remains ferromagnetic with barely unchanged electronic structure up to the Mbar range. As discussed in the introductory part, other Fe-oxides undergo magnetic collapses at lower pressure. The exceptional stability of the magnetic moment in SrFeO$_2$ was studied by first principle calculations^16^. The calculations confirm a transition from a high moment to a low moment configuration at a pressure of $\approx$ 45 GPa related to a crystal field induced spin state transition. At higher pressure, the magnetic moment is found to remain fairly stable with a slight decrease from 2.2 $\mu _B$ to 2.0 $\mu _B$ from 40 to 100 GPa. The predicted behaviour is well in line with our finding although the decrease of the magnetic moment is hard to establish here considering the experimental uncertainty on the XMCD signal. The calculations further predict a faster the decrease of the magnetic moment above 120 GPa to reach 1.2 $\mu _B$ at 200 GPa and a sudden collapse of the magnetic state at 210 GPa which can be linked to the expected second spin state transition^14^. This pressure range is however beyond reach of our current experimental setup and call for further studies at higher pressures. The stability of the Fe electronic properties of SrFeO$_2$ in the Mbar range is ascribed to the increase the FM coupling when atomic distances shortened.

Following the discovery of SrFeO$_2$, another Fe compound belonging to the same family with square planar environment, Sr$_3$Fe$_3$O$_5$, was synthesized^18^. The new compound has a spin ladder structure and shows equivalent sequence of spin transitions under pressure, namely from AFM $S=2$ to FM $S=1$ around 35 GPa, suggesting the generality of the magnetic behavior within the Sr$_{n+1}$Fe$_n$O$_{2n+1}$ family. Following our results in SrFeO$_2$, it would be interesting to confirm the high pressure stability of other compounds of this family in the Mbar range.

Finally, we note that the square planar metal oxide environment has received a considerable attention recently as it is realized in the newly found Ni-based superconductors family^19^. This interest includes compelling pressure effects such as the recently reported increase of $T_c$ in one of these materials^20^.

## Methods

SrFeO$_2$ powder was synthesized at Kyoto University as described in Ref.^12^. For X-ray diffraction, a diamond anvil cell (DAC) was prepared with 100 $\mu$m culet diamonds. A rhenium gasket was indented to reach a thickness of 18 $\mu$m and drilled with a hole of 60 $\mu$m. Powder sample was placed inside, together with gold powder to monitor the pressure using gold known equation of state. We used helium as pressure transmitting medium (PTM), with an initial pressure of 13 GPa. The XRD experiment was performed at PSICHE beamline at Synchrotron SOLEIL. About one hundred diffractograms were collected between 13 and 111 GPa, with a wavelength of 0.3738 Å. Le Bail fit was used on each spectra to extract the *a* and *c* parameters.

For low temperature XAS and XMCD, a CuBe non magnetic DAC was used with 250 $\mu$m culet diamonds. A rhenium gasket was indented at a thickness of 20 $\mu$m, and a 125 $\mu$m hole was drilled. The sample was loaded together with a ruby chip to measure the pressure using the standard ruby fluorescence technique. Neon was used as PTM. The DAC was placed inside a circulating helium cryostat to cool down the cell to 4 K. XAS and XMCD was performed up to 50 GPa at the Fe K-edge ($E_0$ = 7.11 keV) at the ODE beamline at Synchrotron SOLEIL. The energy-dispersive photon beam was focused at the sample region with an elliptically curved Si(111) crystal^21^. To obtain the dichroic spectra, the magnetic field of 1.3 T at the sample position was reversed while keeping a constant polarization of the incident photons. Additional XAS and XMCD measurements at the Fe K-edge were performed at ID12 beamline at ESRF^22^ up to 102 GPa at room temperature. We used a non magnetic DAC equipped with 100 $\mu$m culet diamonds with 17 $\mu$m thick rhenium gasket. The sample powder was loaded with a ruby chip with helium as PTM. The pressure was estimated from the Raman spectra of the diamond tip as the ruby signal is no longer reliable in the Mbar pressure range. For the lower pressure points, a similar DAC equipped with 300 $\mu$m diamond, an 30 $\mu$m stainless steel gasket with 150 $\mu$m hole and helium PTM were used. Ruby fluorescence technique was employed to determine the pressure. At the ODE beamline, the XAS spectra were measured for each pressure point with a fixed polarization at both magnetic field (+ and $-2$ T) and the XMCD extracted directly from the difference. At ID12, to remove systematic errors due to the energy scan, the dichroic signal was extracted from the double difference of the XMCD at $+2$ T and $-2$ T (divided by 2) with both left and right polarizations. All spectra were normalized to unity at high energy using the Athena software and corrected for the circular polarization rate.

## Data Availability

The datasets used and/or analysed during the current study are available from the corresponding author on reasonable request.
